# Calomel Reduced to the Protoxide in the Primæ Viæ

**Published:** 1842-02

**Authors:** 


### CALOMEL REDUCED TO THE PROTOXIDE IN THE PRIMÆ VIAÆ.

Dr. Runyon, of Trenton, Tennessee, writes to us that he has obtain ed globules of mercury as large as a squirrel shot, from the black matter discharged by some of his patients under the action of calomel. The matter is called "black sand," and resembles "pulverized charcoal." This deposition he proves to be the protoxide of mercury, by rubbing it in a mortar with magnesia, when the globules of quicksilver appear.

In such cases, Dr. R. says he is in the habit of combining opium with the calomel; but as this article is sometimes contra-indicated, he wishes to know whether any other substance can be conjoined with the calomel which will prevent it from undergoing this change in the primæ viæ. If any of our readers have been furnished by their experience with such a remedy, they will confer a favor on Dr. R. by communicating it. Y.

